# A Sitting Posture Monitoring Instrument to Assess Different Levels of Cognitive Engagement

**DOI:** 10.3390/s19030455

**Published:** 2019-01-22

**Authors:** Daniele Bibbo, Marco Carli, Silvia Conforto, Federica Battisti

**Affiliations:** Department of Engineering, University of Roma Tre, Via Vito Volterra, 62, 00146 Rome, Italy; marco.carli@uniroma3.it (M.C.); silvia.conforto@uniroma3.it (S.C.); federica.battisti@uniroma3.it (F.B.)

**Keywords:** posture, cognitive engagement, stress level, pressure sensors, embedded systems, body expression, sensorized seat

## Abstract

An office chair for analyzing the seated posture variation during the performance of a stress-level test is presented in this work. To meet this aim, we placed a set of textile pressure sensors both on the backrest and on the seat of the chair. The position of the sensors was selected for maximizing the detection of variations of user’s posture. The effectiveness of the designed system was evaluated through an experiment where increasing stress levels were obtained by administering a Stroop test. The collected results had been analyzed by considering three different time intervals based on the difficulty level of the test (low, medium, and high). A transition analysis conducted on postures assumed during the test showed that participants reached a different posture at the end of the test, when the cognitive engagement increased, with respect to the beginning. This evidence highlighted the presence of movement presumably due to the increased cognitive engagement. Overall, the performed analysis showed the proposed monitoring system could be used to identify body posture variations related to different levels of engagement of a seated user while performing cognitive tasks.

## 1. Introduction

Nowadays, a large percentage of the active population is spending many hours sitting either for work or leisure (office workers, watching TV, etc.). Recent studies have shown that an increased sitting time may induce chronic diseases (eventually leading to death) [1] and may also have a severe impact on psychological health (leading to anxiety and depression) [2].

To mitigate these effects, in recent years many solutions have been proposed for reducing sitting time. Among these, it is worth mentioning the use of height-adjustable sit–stand workstations. As shown in [3], their use not only decreases sitting time but also reduces the cardio-metabolic risk. Another option for avoiding prolonged static postures is to perform micro-breaks every 20–30 min of continuous sitting [4]. Nowadays, there are several options to support the implementation of these breaks. Among them, the use of traditional alarms, computer prompts (e.g., Stretchly [5], Big Stretch Reminder [6], Awareness [7]), smart watches (e.g., Apple Watch, Fitbit trackers) and mobile applications (e.g., Break Timer [8], Eye Care 20 20 20 [9]) are a few examples.

Despite these options, the time spent sitting is increasing, as analyzed in [10,11,12]. Knowledge of the posture of a seated person turns out to be useful in many ways.

One of the main applications of the detection and classification of posture is in the medical field—by being able to detect that a person is sitting in a wrong position, it is possible to correct it and reduce its negative consequences on health. It is useful to underline that adopting a correct sitting posture is not easy for all people. In fact, it is common to adopt, during everyday activities, a wrong posture even if a correct ergonomic seat is provided; for example, sitting slumped apparently is a more comfortable position than sitting with the back straight, but over time, it may cause tension, with muscle or joint pain [13]. Among other negative consequences, a wrong posture may lead to acute or chronic problems, such as back pain [14], headaches [15], or digestive problems [16]. Even if posture detection is extremely useful in health monitoring and rehabilitation, as well as human movement dynamic analysis [17], other applications can benefit from this technique—design of ergonomic chairs [18,19], virtual reality applications [20], or teaching and learning environments [21].

In the literature, several approaches have been proposed to implement automatic systems to analyze the posture of a seated person, usually indicated as sitting posture monitoring systems (SPMS). The main purpose of these systems is either checking the presence of a person or identifying his/her position.

Some of the proposed solutions relied on the use of wearable sensors [22,23,24,25], while others exploited non-invasive approaches in which the sensors were applied on the environment (e.g., chairs [26,27,28,29,30,31,32,33]). In this way, participants were not aware of being monitored, thus allowing the reproduction of real-life conditions.

Roh et al., in [34], implemented an instrumented chair by inserting four load cells in the seat frame of an office chair and tested the capability of the proposed system by using machine learning techniques for the automatic classification of sitting postures. The use of load cells may provide accurate results, but it required periodic and accurate calibration.

A similar approach was presented in [33], where five machine learning algorithms were used to classify posture position. The features used for training and testing these algorithms were the outputs of 16 pressure sensors located on the seat, on the backrest, and on the armrests of the office chair. Aiming at a basic posture classification, the system seemed to be complex and expensive.

Ishac et al., in [29], presented a cushion made of a conductive fabric pressure sensing array. The cushion was placed in the backrest of the chair and it allowed recording the pressure on nine different points. The lack of a seat pressure map provided limited information of the body posture.

As can be noticed, the interest of the research has been mainly focused in the detection and classification of the user’s posture. Few efforts have been devoted to the high-level analysis of the data collected through SPSMs that can be exploited to extract information on users’ behavior. An example is in [35], where the authors analyzed the variation of the center of pressure of sitting participants that were in a condition of fatigue and return to a state of non-fatigue. The outcomes of this research highlighted that the user’s posture was the most stable when the user was tired, thus concluding that fatigue could be inferred from a sensorized chair.

In the literature, the problem of inferring an emotional status from the sitting position was addressed in [31,32]. As far as we know, no existing works investigated the onset of stress, while in many working scenarios (i.e., control room), the monitoring of the stress level of an operator was very important for preventing human errors or misjudgments.

Based on a previous work [36], the aim of the present study is to investigate if the variation of the sitting posture is influenced by the stress level driven by an increasing cognitive engagement.

## 2. Materials and Methods

### 2.1. Smart Chair

**Figure 1:**
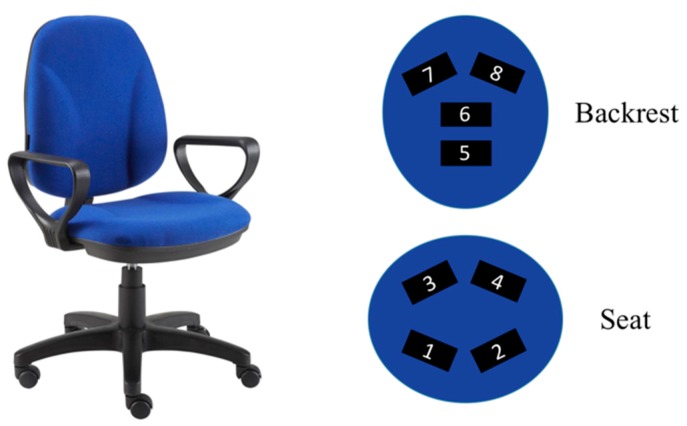
The smart chair and the sensors’ position on the chair—analog tactile pressure sensors (ATSs) 1–4 are located on the seat while ATSs 5–8 are located on the backrest.

To monitor different human body postures over time, a common office chair had been equipped with pressure sensors. In the design of the chair, this kind of sensor had been selected due to its dimension and shape—the use of a non-invasive system was very important in order not to interfere with normal seated posture while providing the same comfort to the user. While seating in a correct position, two areas of the body were in contact with the chair—the pelvis together with upper shanks and the back. The contact points of these body areas usually changed with time. However, it was possible to identify some sub macro areas that, in normal conditions, were always in contact with the chair—e.g., while seated, the participant may “push” more with the pelvis or the shanks, on the right or left part of the seat. At the same time, the participant may place on the rear, on either the right or the left side, different parts of the back (e.g., only the lower part, only the upper part, all the back). These patterns could be monitored using two sets of pressure sensors placed on the seat and on the chair backrest. The designed chair was equipped with analog tactile pressure sensors (ATS, PW073/PW074, Plug&Wear, Italy), made of stainless steel microfibers, polyester, cotton, and mostly polyethylene tissue. Each ATS looked like a soft tissue that did not change the chair upholstery features when fixed on it. The use of conducting fibers in the fabric also allowed measuring pressure distribution in uneven surfaces. Furthermore, ATSs presented high flexibility and extensibility, together with a limited size, thus allowing a complete integration in the chair upholstery with no impact on the user experience. In the designed chair, two sets of four ATSs are placed below the fabric, as shown in Figure 1.

The designed configuration allowed monitoring of all the pelvis positions described above (i.e., along both the anterior-posterior and the medio-lateral directions) together with the partial or complete leaning back, through the analysis of each ATS activation status. An ATS is assumed active when its level of pressure overcomes a defined threshold. 

**Figure 2:**
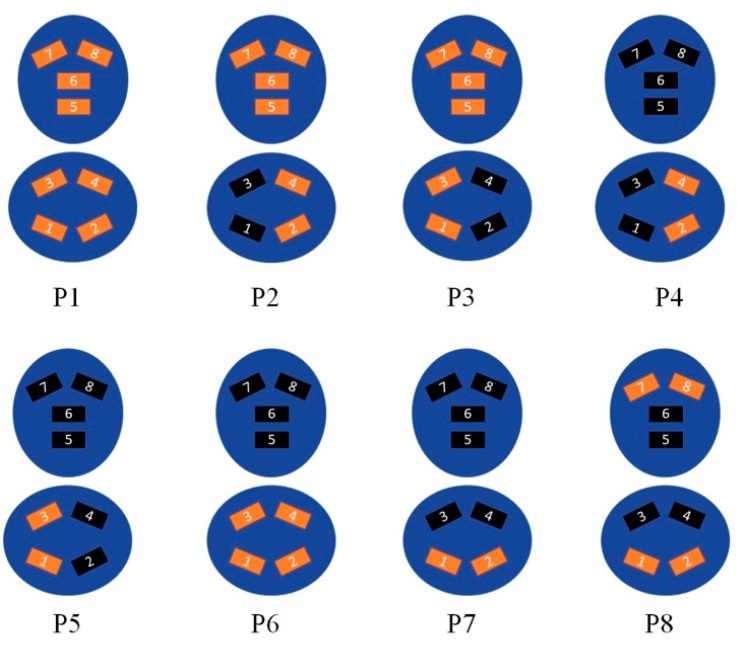
Different ATS combinations. The red rectangles indicate the activated ATSs.

Based on different active ATSs combinations, shown in Figure 2, specific postures are recognized as described in Table 1.

### 2.2. Acquisition System

**Figure 3:**
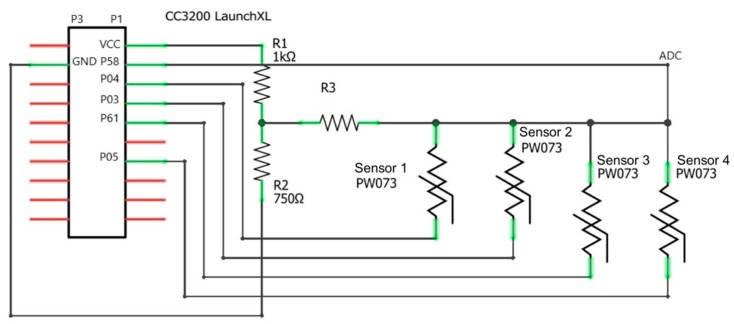
Electrical sketch of the sensors’ configuration used for the seat set. The same scheme is adopted for the chair back.

To collect signals from ATSs and to provide the smart chair with processing and delivering capabilities, an embedded platform based on a microcontroller unit (MCU) was used (i.e., Texas Instruments CC3200 LaunchXL). This platform had been specifically designed for the use in the Internet of Things (IOT) applications. It was provided with a CC3200 Wi-Fi microcontroller, a 4-Wire JTAG and 2-Wire SWD programming interface, and a USB interface to PC for CCS/IAR using FTDI USB drivers, and it can be powered by 2 AA alkaline batteries. With the aim of optimizing the electronic resources in the chair design, only 2 A/D channels, one for each ATS set, are used, as depicted in Figure 3.

This configuration allowed limiting the power consumption while providing left A/D channels for other sensors. Each ATS was powered by a digital input/output pin (DI/O) of the MCU and all ATSs were short-circuited to the same ADC pin 58. A voltage divider was used to limit each ATS power range, as required by the datasheet. The sampling frequency was set to 45 Hz, based on the frequency content of the ATS’ signal. Each ATS was enabled by switching its corresponding DI/O pin from the input to the output status and setting it to the value “low”. ATSs were enabled one at a time with a duty cycle lower than the sampling period. Therefore, the four corresponding signals were acquired by using only one ADC channel. To do this, a specific algorithm was implemented, and then deployed to the onboard system, using the Texas Instruments Energia framework, including the digitalized data transmission through the USB/serial connection. Data collection was performed through a user interface developed with Matlab (@The Mathworks, Inc.) for handling the USB/serial connection.

### 2.3. Experimental Setup

To validate the effectiveness of the smart chair for monitoring the participant’s sitting behavior while a stressing situation occurred, a specific test (i.e., the Stroop test) had been designed and conducted on a population of volunteers. In detail, the values of the sensors collected while the user was performing the test were analyzed and processed to predict the position of the participant. The information on the position was then used for inferring the stress level.

#### 2.3.1. Stroop Test

The performed test was based on the Stroop effect that involved asking a participant to perform a specific cognitive task in a predefined time slot to elicit a feeling of stress [37]. The correlation between the performance of the Stroop test and the increase in the stress level was discussed in [38]. The authors recorded several parameters (e.g., heart rate, skin conductance responses, and self-reported anxiety) of participants performing a Stroop test. Similarly, in [39], the authors analyzed the stress induced by the performance of the same test by using the electrocardiographic and the heart rate variability signals. The results of the previous studies highlighted the presence of a significant variation in these signals between the stressed and the normal conditions.

The test performed in this study was designed by exploiting the Stroop effect for inducing different stress levels in the participants. During the test, some words were shown to the participant—the words were the name of a color (e.g., “blue”, “red”, “black”, etc.) and were presented written in a colored form. The participant should name the color of each word, independently from the meaning of the word. The possible combinations were the following: (i) the name and the color were congruent (e.g., the word ”red” was printed in red); (ii) the name and the color were not congruent (e.g., the word ”red” was printed in green). The first case usually represented a simple task, while the second one, to be completed, would require more time than what was allowed, thus inducing the participant to make errors (e.g., naming the word and not the color of the word or not reading the complete set of words).

**Figure 4:**
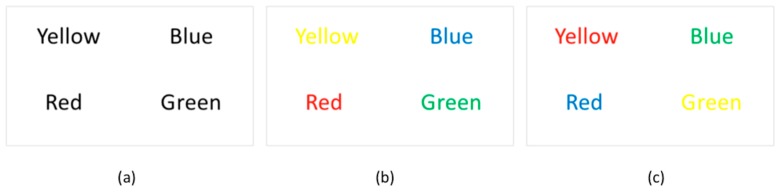
Familiarization color names (**a**); congruent color names (**b**); and non-congruent color names (**c**) displayed during the complete Stroop test.

Following this paradigm, a Stroop test composed by 25 slides had been prepared to be shown to the participants seated on the instrumented chair. In the first part of the test, 6 slides containing color names written in black were shown to make the participant familiar with the task of reading words (Figure 4a). Then, in the second part, 6 slides containing names congruent with their color were shown to the participants (Figure 4b). In the last part of the test, 12 slides containing names not congruent with their colors were shown to the participants (Figure 4c). The font size adopted for all displayed words (96 pt.) was the same over the test duration and the slides were displayed in full screen mode. The number of words increased slide by slide, while the time transition between consecutive slides was fixed for providing uniform testing conditions for all participants across the different tasks. The limited time-length constraint was introduced to further induce stress in the participants while the Stroop effect occurred, because participants would need more time to complete the second part of the experiment with no errors. The adopted settings are reported in Table 2.

#### 2.3.2. Experimental Protocol

Before starting the test, participants were invited to sit on the chair with a comfortable and natural posture. They were asked to sit without crossing their legs and lying on the back, which was the posture denoted as P1. No information about the smart chair or the presence of sensors was given to participants prior the test to avoid bias in participants’ behavior. The test started with a brief displayed introduction containing instructions (IS1) about the task to be performed. In this way, time to find the most comfortable position was given to each participant. More instructions (IS2) were given at about one-third of the test, when colored words were displayed on the monitor. The given instructions consisted of (IS1) to read aloud each word appearing on the slide and (IS2) to read and to pronounce aloud the name of the color of the displayed words as quick as possible, without considering the meaning of the word. The length of the test was set to less than two minutes. At the end of the test, the participants were informed about the data recorded during the experiment (i.e., pressures or timing) and that the test was part of a scientific study. Furthermore, to verify the possible perception by the user about the presence of sensors, participants were interviewed at the end of each session. For all participants, the chair provided the same level of comfort as a normal office chair. They were asked to give their consent to the use of the recorded data, for the finality of this study, together with the information on gender and age only. The tests had been performed on a population of 114 participants (69 males and 45 females), with age (mean ± SD) of 32 ± 12. All volunteers accepted for performing the test were healthy with no apparent or declared mobility problems (e.g., use of walking aids) or visual impairments (i.e., none of them wore glasses or contact lenses, all volunteers were checked for color blindness). Another requirement was to be a native Italian speaker, since the slides were prepared using only Italian words. The participants were recruited during the “Maker Faire 2017” (The European edition, Rome, Italy) among the population visiting the exhibition.

### 2.4. Data Elaboration and Analysis

In order to classify different postures, ATSs activations were evaluated and compared to identify one of the possible combinations described in Section 2.1. These pressure sensors returned a value proportional to the load applied. A threshold value was set, as described in the next paragraph, to define whenever the ATS was active. To reduce the noise present in the signal and to better evaluate the trend variation over the test duration, a moving average filter, with a cutoff frequency of 1 Hz, was used for each ATS output. Finally, the data collected over time were compared to understand how the participant’s behavior changed during the test. The analysis was performed by using Matlab (@The Mathworks, Inc.) on the adopted postures by all participants during the tests. To better understand the posture variation of participants, three different time intervals had been identified:

- TI1: Time interval when words were displayed in black and words and colors were congruent.
- TI2: Time interval when words and colors were not congruent, with a small number of words (3–6).
- TI3: Time interval when words and colors were not congruent, with a large number of words (9–12).

In this way, intervals with an increasing level of engagement, and consequently with a rising level of stress were identified.

#### 2.4.1. Adaptive Threshold Determination

In order to obtain correct information on the sensor activation, signals were processed considering the baseline level for each test. Each sensor provided a different value when no load was applied and when pushed by different participants. Therefore, it was not possible to fix a predetermined threshold. To cope with this problem, a setup phase had been performed considering that a generic sensor had a non-linear and inverted behavior—it gave a maximum voltage output when no load was applied and approached a minimum one when it was pushed with an increasing loading action. To set these limits, before each test a preliminary acquisition with no load applied was performed and the mean value *Vzero* was extracted from each ATS. Then, the sensors’ signals were acquired with the participant initially seated in P1. In this way, the condition for all ATS being active was reached, corresponding to the minimum voltage values *Vmaxload*. Finally, the threshold used for each test was set as the 50% of the range between *Vmaxload* and *Vzero*.

#### 2.4.2. Transition Analysis

To understand how postures change in the time intervals of the test (TI1, TI2, and TI3), a transition analysis was performed between TI1–TI2 and TI2–TI3. The aim was to identify the starting postures in TI1 (or TI2) leading to a new posture in TI2 (or TI3). Moreover, considering the directions of transitions, it was possible to understand if these were monotonic (i.e., transitions between pairs of postures occurred only in one direction).

Contingency tables had been used to implement the transition analysis and, in particular, to evaluate the associations between test administration and postures. These tables were compiled considering the two transitions TI1–TI2 and TI2–TI3 and counting the number of participants changing their posture during the test administration. Chi-square statistics were used to test the significance of associations, by implementing a McNemar test [40] for multiple paired data and the McNemar–Bowker test to apply the global test for symmetry [41,42]. A value of 0.05 < *p* < 0.10 indicated an interest to note but not a compelling evidence, while *p* < 0.05 denoted a statistical significance.

The complete transition analysis, i.e, the TI1–TI2–TI3 had been carried out only in a descriptive way because the data sample was not sufficiently big to undergo a statistical analysis (the number of constraints in the statistical analysis was 8 × 8 × 8 which was greater than the number of data).

## 3. Results

The data collected had been analyzed by considering both the behavior of the participants and the outputs of the sensors. From the behavior observations, a postural modification between the first and the last part of the test was noticeable for all participants. The task performed in the initial part was very easy, and the participants were able to maintain the initial position. When the task difficulty increased, such as during non-congruent word and reduced time interval slides, the participants had the tendency either to lean forward for better focusing on the test or to push him/herself away from the screen. These observations were confirmed by the data acquired by the sensors.

**Figure 5:**
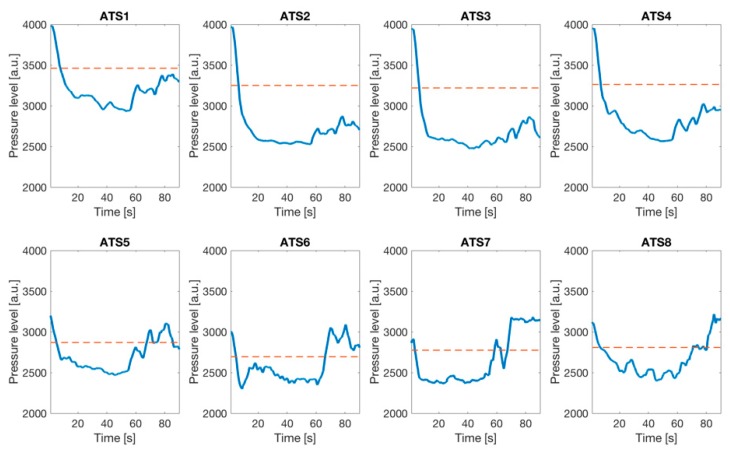
ATSs pressure trend over time for one of the participants during the experiment. If the signal is under the threshold (dashed line), the sensor is pressed.

In Figure 5, the time trends of the ATSs output is reported for one participant as an example. The general trends of the sensors on the seat (ATSs 1–4) showed a pressure level that generally remained under the threshold, while the general trends of the sensors on the backrest (ATSs 5–8) showed a pressure level that generally increased during the experiment. At the beginning of the test, the combination of the pressure levels of ATSs identified a posture in which the participant was sitting and leaning back on the backrest (i.e., P1). The pressure combination at the end of the test indicated that the participant was bending forward toward the screen moving to a different posture (i.e., P6), thus denoting a variation with increasing cognitive engagement.

The algorithm for posture identification processed all the properly acquired signals. Recordings showing failures of the sensors were discarded, thus reducing the dataset to 92 participants. The identification succeeded completely in the three time intervals for 83 participants, while in nine cases the active ATSs combinations were not recognized as one of the eight postures taken into account in this study.

**Figure 6:**
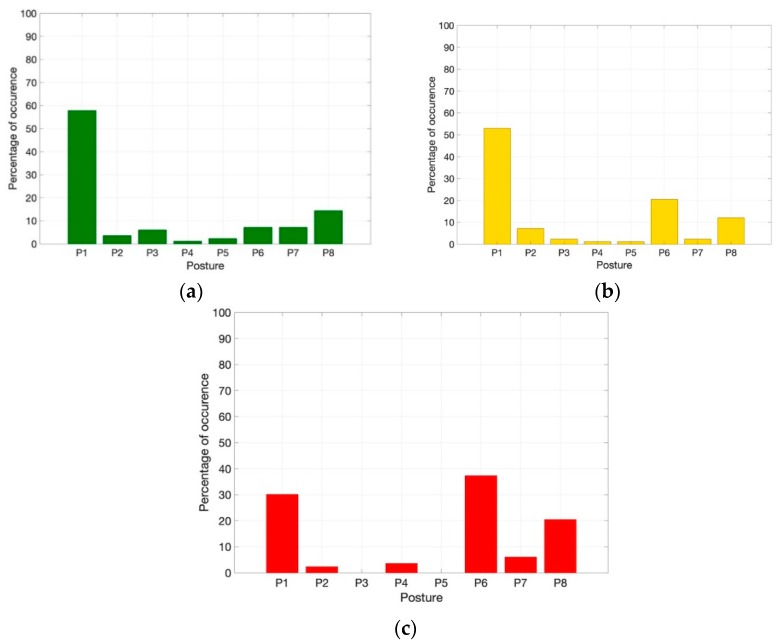
Distributions of identified postures for all participants in (**a**) TI1; (**b**) TI2; and (**c**) TI3.

In Figure 6 the distributions of the identified postures for all the analyzed participants in the three intervals TI1, TI2, and TI3 are reported.

In the TI1 interval (Figure 6a), 59% of the participants adopted posture P1 while 7%, 7%, and 14% were in postures P6, P7, and P8, respectively.

In the TI2 interval (Figure 6b), 53% of the participants adopted posture P1, while the combined adoption of postures P6, P7, and P8 increased with respect to TI1 (i.e., 28 to 36%).

In the TI3 interval (Figure 6c), a reduced number of participants adopted posture P1 (30%), and the majority of the participants were in postures P6, P7, andP8 (64%).

The results of the contingency table analysis are reported in Table 3 for transition TI1–TI2, and in Table 4 for transition TI2–TI3. In both tables, rows represent postures adopted in the first time interval of the transition (i.e., TI1 in transition TI1–TI2 or TI2 in transition TI2–TI3), while columns represent postures adopted in the final time interval of the transition (i.e., TI2 in transition TI1–TI2 or TI3 in transition TI2–TI3). Each cell {*i*,*j*} (*i* = 1,…,8; *j* = 1,…,8) contains the number of participants who move from posture *i* to posture *j*. The cells situated in the main diagonal contain the number of participants who did not change posture.

The transition analysis for TI1–TI2 (Table 3) showed that the largest number of participants remained in P1 (31), while 9 participants moved from P1 to P6 and 7 from P8 to P1. The other transitions were executed by a number of participants lower than 4.

The transition analysis for TI2–TI3 (Table 4) showed that 14 participants remained in P1 and 6 participants remained in P6. Transitions between different postures were the following—21 participants moved from P1 to P6, 7 participants moved from P1 to P8, 7 participants moved from P6 to P8, 4 participants moved from P6 to P1, and 4 participants moved from P8 to P1. The other transitions were executed by a number of participants lower than 3.

Moreover, some of the participants did not change their posture during the entire test—14 participants did not move from P1, 2 participants did not move from P6, and 2 participants did not move from P8.

The results of the McNemar test for multiple paired data, performed considering all possible posture pairs, give the *p*-values reported in Table 5 and Table 6 for transitions TI1–TI2 and TI2–TI3, respectively. The different posture pairs underwent the test only if the McNemar condition [40] was respected.

The results showed that for TI1–TI2, all the transitions undergoing the test were not statistically significant neither for *p* < 0.1 nor for *p* < 0.05; for TI2–TI3, the results showed that the transition P1–P6 was significant for both *p* < 0.1 and *p* < 0.05, while the transition P6–P8 was significant for *p* < 0.1.

The global test for symmetry made by the McNemar–Bowker analysis showed a *p*-value = 0.083 for the transition TI1–TI2 and a *p*-value = 0.0002 for the transition TI2–TI3.

**Figure 7:**
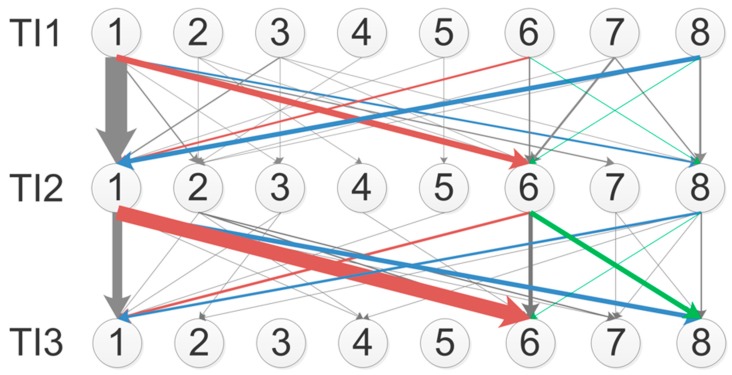
Descriptive analysis of the two-step transition TI1–TI2–TI3 (colored edges highlight the main associations between pairs of postures).

The descriptive analysis of the two-step transition TI1–TI2–TI3 is reported in Figure 7, where an oriented graph is reported. The graph topology used three layers representing the time intervals TI1, TI2, and TI3; each layer was composed by eight nodes representing the adopted postures. The edges from the first to the second layer and from the second to the third layer represented the transitions TI1–TI2 and TI2–TI3, respectively. The edges thickness was proportional to the number of transitions. The graph highlighted the presence of some preferential transitions and that postures P1, P6, and P8 were the most populated (those highlighted by the colored edges) with P1 being preferred in the initial phase of the test and P6 and P8 being chosen at the end of the test.

**Figure 8:**
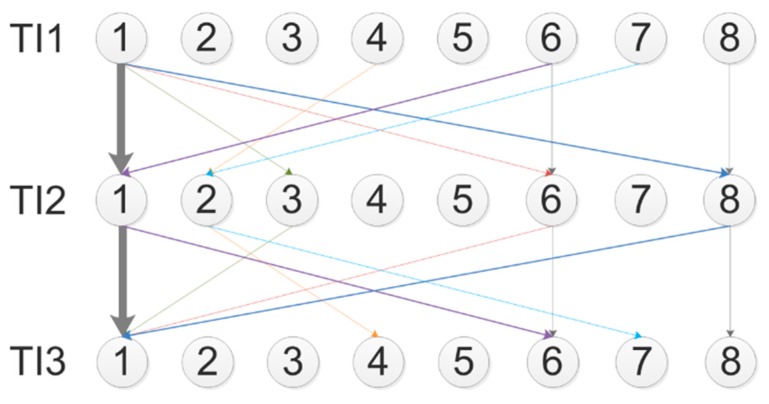
Descriptive analysis of the two-step transition TI1–TI2–TI3 for no-transitions (grey edges) and non-monotonic transitions (colored edges).

From the previous graph, some data have been extracted regarding only the participants who either maintained the same posture over the time intervals (no-transitions, 13 out of 83) or moved back to the original posture (non-monotonic transitions, 8 out of 83). Those data are displayed in Figure 8, using an oriented graph having the same topologies and characteristics of the one in Figure 7.

Postures P1, P6, and P8 were the only ones characterized by no-transitions. A limited and fragmented amount of non-monotonic transitions was noticed.

## 4. Discussion

The proposed system can be used to give insights on the level of cognitive engagement of participants and their stress level, as it has been demonstrated by the above results. Since the sensors were hidden in the cushions of the chair, users were not able to perceive the difference with a traditional one, so the presented system was non-invasive. This feature was of primary importance during the tests to avoid bias in participants’ behavior.

In detail, the results for the three considered time intervals showed a change in the adopted sitting postures.

In the TI1 interval, with the first level of engagement in the Stroop test (Figure 6a), the posture P1, suggested by the experimenters as the initial one, was adopted by most of the participants who confirmed with this behavior the comfort of P1. It is worth noticing that a non-negligible number of participants assumed P6, P7, and P8. This can be interpreted considering that the first part of the test was used by the participants to get familiar with the task to be performed and to find the most comfortable position on the chair, and that for some participants this can be different from P1.

In the TI2 interval, by increasing the level of engagement (Figure 6b), 53% of participants were in posture P1, while the number of participants adopting postures P6, P7, and P8 (23%, 2%, and 11%, respectively) increased with respect to TI1, thus showing that different postures on the chair started to be adopted. Moreover, in this time interval, the widening of the range of the adopted postures suggested an increased level of engagement for some volunteers.

In the TI3 interval (Figure 6c), a minority of participants remained in posture P1 (30% of adopted postures), and the majority of participants adopted postures P6, P7, and P8 (respectively, 36%, 5%, and 23%), thus confirming that with a high engagement in the test, the majority of the participants tend to lean forward on the chair for better focusing on the test (P6, P7) or to push him/herself away from the screen, as to escape from the stressing situation (P8).

The analysis of the contingency table over the time interval TI1–TI2 (Table 3) showed that a considerable number of participants remained in P1, thus denoting a low effect of the first part of the Stroop test on the posture variation. The low difficulty level of this part of the test did not induce in these participants a consistent level of stress driving a movement on the chair. Some non-negligible transitions showed up from P1 to P6 and from P8 to P1, even if they were not statistically significant when considered as transition pairs (*p* = 0.149 and *p* = 0.343, respectively). These transitions could be interpreted as a first sign of discomfort due to the increased level of engagement. The test for global symmetry, with a *p*-value = 0.083, demonstrated that globally the postures distribution had changed, thus highlighting the effect of the increased level of engagement.

The analysis of the contingency table over the time interval TI2–TI3 (Table 4) showed that the number of participants remaining in P1 was halved, thus denoting that the increasing level of engagement of the Stroop test pushed the participants to move away from P1. P6 and P8 became intensively populated. The statistical test demonstrated the significance for transitions P1–P6 (*p* = 0.001) and P6–P8 (*p* = 0.077) highlighting that P6 and P8 assumed importance with the increased level of engagement and started to replace P1 as the preferred posture. The test for global symmetry, with a *p*-value = 0.0002, demonstrated that the postures distribution had significantly changed with a higher level of engagement induced by the Stroop test.

The oriented graph showing the global transitions, reported in Figure 7, is presented for descriptive purposes only, since a statistical validation on this two-stage transition model is prevented from the limited number of data. An element of interest to be discussed relates to differences between monotonic and non-monotonic transitions. The occurrence of non-monotonic flows is not evident from the description in Figure 7. However, to go in deeper details, a further graph (Figure 8) has been reported where only the no-transitions and the non-monotonic transitions are reported. With this graph, the idea of neglecting non-monotonic transitions is confirmed. This is in line with the experimental design in which the test is administered by adopting increasing levels of engagement—non-monotonic flows would imply a return to the initial posture associated with the level of engagement used at the beginning of the experiment, which is not the case of this study. Considering that the number of no-transitions for P6 and P8 is just one for both, and that the number of non-monotonic transitions affects P6 for only two participants, it shows up that adopting postures P6 and P8 at the end of the experiment is due to the occurrence of monotonic transitions induced by the Stroop test.

## 5. Conclusions

In this contribution, a new system to evaluate the posture of a seated person is presented. The tests demonstrate that this system can be used in situations whenever different level of engagement, that can drive different levels of stress, occur. In fact, the different levels of engagement produce, in general, a different positioning on the chair of the participants, that moved from a comfortable and natural posture to others indicative of an increased difficulty in performing the required task. The designed system is based on the use of simple textile sensors and of low-cost electronic equipment that can be easily embedded in the chair structure. This, combined with a wireless transmission, can delineate a final product that could be part of a work environment, in which stressing situations can occur for different and not expected reasons, thus increasing the level of safety.

All the analyses in this study have not taken in account the dynamic parameters of the posture—in fact, the selected sensors were only used as a “switch” (i.e., considering an ATS active or not), to maximize the reliability of the results. In future development of the chair features, more advanced sensors can be adopted, as strain gauges based ones or sensors measuring the rotation of the chair, in order to obtain signals that can be used to extract dynamic parameters proportional to the level of pressure applied or to the swinging movement. This data may also provide information regarding the movement of the participant in different tasks.

## Figures and Tables

**Table 1:** Posture identification and description.

Posture	Name	Description	Active ATS							
			1	2	3	4	5	6	7	8
P1	All	All sensors of the chair are uniformly pressed	1	1	1	1	1	1	1	1
P2	OverLapRightBack	Right leg crossed and sensors positioned on the chair backrest uniformly pressed	0	1	0	1	1	1	1	1
P3	OverLapLeftBack	Left leg crossed and sensors positioned on the chair backrest uniformly pressed	1	0	1	0	1	1	1	1
P4	OverLapRight	Right leg crossed and the participant is not leaning on the chair backrest	1	0	1	0	0	0	0	0
P5	OverLapLeft	Left leg crossed and the participant is not leaning on the chair backrest	0	1	0	1	0	0	0	0
P6	SeatAll	Sensors positioned on the seat are uniformly pressed and the participant is not leaning on the chair backrest	1	1	1	1	0	0	0	0
P7	SeatFront	The participant uses only the front portion of the seat and is not leaning on the chair backrest	1	1	0	0	0	0	1	1
P8	SeatFrontBackUp	The participant uses only the front portion of the seat and the upper part of the chair backrest	1	1	0	0	0	0	1	1

**Table 2:** Schedule of the Stroop test—in the first phase the color of words corresponds to the word, while in the second part the words are written in different colors.

	Number of Words Per Slide	Number of Slides	Time Per Slide [s]
Words are displayed in black	3	2	2
	4	2	2.5
	5	1	3
	6	1	3.5
Words and colors are congruent	3	2	2
	4	2	2.5
	5	1	3
	6	1	3.5
Words and colors are not congruent	3	2	2.5
	4	2	3
	5	2	3.5
	6	2	4
	9	2	6
	12	2	8

**Table 3:** Transition analysis over time interval TI1–TI2.

	P1	P2	P3	P4	P5	P6	P7	P8
P1	31	2	1	0	0	9	2	3
P2	0	1	0	1	0	1	0	0
P3	2	0	1	0	0	1	0	1
P4	0	1	0	0	0	0	0	0
P5	1	0	0	0	1	0	0	0
P6	3	0	0	0	0	2	0	1
P7	0	1	0	0	0	3	0	2
P8	7	1	0	0	0	1	0	3

**Table 4:** Transition analysis over time interval TI2–TI3.

	P1	P2	P3	P4	P5	P6	P7	P8
P1	14	0	0	1	0	21	1	7
P2	1	0	0	1	0	2	2	0
P3	1	1	0	0	0	0	0	0
P4	0	0	0	0	0	1	0	0
P5	1	0	0	0	0	0	0	0
P6	4	0	0	0	0	6	0	7
P7	0	0	0	0	0	0	1	1
P8	4	1	0	1	0	1	1	2

**Table 5:** *p*-values for the McNemar test performed on the TI1–TI2 transition.

	*p*-value
P1–P3	1
P1–P6	0.149
P1–P8	0.343
P2–P4	0.479
P6–P8	0.479

**Table 6:** *p*-values for the McNemar test performed on the TI2–TI3 transition (* *p* < 0.1, ** *p* < 0.05).

	*p*-value
P1–P6	0.001 **
P1–P8	0.546
P6–P8	0.077 *
P7–P8	0.479
